# TM4SF4 overexpression in radiation-resistant lung carcinoma cells activates IGF1R via elevation of IGF1

**DOI:** 10.18632/oncotarget.2450

**Published:** 2014-09-08

**Authors:** Soo-Im Choi, Seo-Yeon Kim, Jaeha Lee, Eun-Wie Cho, In-Gyu Kim

**Affiliations:** ^1^ Department of Radiation Biology, Environmental Radiation Research Group, Korea Atomic Energy Research Institute (KAERI), Daedeok-daero, Yuseong-gu, Daejeon, South Korea; ^2^ Department of Radiation Biotechnology and Applied Radioisotope, Korea University of Science and Technology, Daedeok-daero, Yuseong-gu, Daejeon, South Korea; ^3^ Epigenomics Research Center, Korea Research Institute of Bioscience and Biotechnology (KRIBB), Gwahak-ro, Yuseong-gu, Daejeon, South Korea

**Keywords:** TM4SF4, lung adenocarcinoma, IGF1R activation, IGF1

## Abstract

Transmembrane 4 L six family member 4 (TM4SF4) is a member of the tetraspanin L6 domain family. Other members of this family, TM4SF1 (also known as L6-Ag) and TM4SF5, have been shown to be upregulated in multiple tumors and involved in epithelial-to-mesenchymal transition and cell migration. However, unlike its homologs, little is known about TM4SF4. Here, we show that TM4SF4 was highly expressed in radiation-resistant lung adenocarcinoma cells, such as A549 and Calu-3 cells, and its expression activated cell growth, migration, and invasion. Overexpression of TM4SF4 in A549 cells increased the activation of PI3K, AKT, and NF-kappaB and the expression of PTEN. IGF1R was clearly activated by overexpression of TM4SF4, although EGFR was also slightly activated. TM4SF4 expression was correlated with the increased expression of IGF1, consequently resulting in IGF1R activation. Tumorigenic activity of TM4SF4 in lung adenocarcinoma cells was also demonstrated by xenograft assay; however, this activity was almost completely suppressed by treatment with anti-TM4SF4 antibody. Our results suggest that TM4SF4 overexpression in lung carcinoma cells results in resistance to radiotherapy via IGF1-induced IGF1R activation and blocking the activity of TM4SF4 using specific antibody can be a promising therapeutics against TM4SF4-overexpressing lung adenocarcinoma.

## INTRODUCTION

Transmembrane 4 L six family member 4 (TM4SF4) is a member of the tetraspanin L6 domain family [1], which includes TM4SF1/L6, TM4SF4/IL-TMP, and TM4SF5/L6H. TM4SF1 and TM4SF5 were originally identified as tumor-associated antigens [2, 3], and their overexpression was reported in multiple tumors, including lung, breast, colon, prostate cancer, and hepatocellular carcinoma [2, 4-8]. TM4SF1 and TM4SF5 affect migratory mechanisms crucial to cancer invasion and metastasis [2, 4, 9-11], making them as crucial targets for cancer therapy [10, 12]. Also, TM4SF1 is defined as a cancer stem cell marker [13], and TM4SF1 and TM4SF5 are reported to be involved in epithelial-to-mesenchymal transition [7, 14], which is also associated with stemness properties [15].

TM4SF4 was originally cloned from intestinal epithelium and liver; for this reason, it was named intestine and liver tetraspan membrane protein (IL-TMP) [16, 17]. TM4SF4 is a 202-amino acid membrane protein that contains four hydrophobic transmembrane domains and two hydrophilic regions [1]. It is classified as a more divergent member of the tetraspanin L6 domain family, owing to a lack of characteristic cysteine residue motifs in the EC2 extracellular domain, and has 50% sequence identity with L6 protein TM4SF1[1]. TM4SF4 levels appear to increase when non-dividing epithelial cells differentiate and migrate out of intestinal crypts [16]. Likewise, in the liver, TM4SF4 is expressed in non-dividing hepatocytes that retain high proliferative potential in the presence of the appropriate stimulus, and is upregulated during liver injury [17, 18], which implies that its functions are related to cellular differentiation or proliferation. However, little is known about functions of TM4SF4 in cancer cells. Only recently, it was reported that *TM4SF4*/*IL-TMP* mRNA and protein levels were upregulated in 80% of hepatocellular carcinoma tissues [19].

Lung cancer is a lethal cancer in both men and women. Non-small cell lung cancer (NSCLC) comprises the majority (greater than 75%) of lung cancers and, when clinically extensive, it is typically characterized by inexorable disease progression despite treatment with chemotherapy and/or irradiation [20]. Because chemotherapy and irradiation induce programmed cell death, or apoptosis, recent efforts have been made to understand molecular events that confer therapeutic resistance. Based on these efforts, the phosphatidylinositol-3-kinase (PI3K)/protein kinase B (AKT) pathway [21] and the IGF1/IGF1R signaling pathway [22] have emerged as potential determinants of radiation resistance in human lung cancer cells.

Here, we show that TM4SF4 is highly expressed in radiation-resistant lung adenocarcinoma cells, such as A549 and Calu-3 cells, and its expression activates cell growth, migration, and invasion via IGF1R activation. Overexpression of TM4SF4 elevated the level of IGF1 induction, which resulted in IGF1R activation and radiation resistance. Treatment of TM4SF4-overexpressing lung carcinoma cells with anti-TM4SF4 antibody suppressed cell growth, which was mediated by suppression of IGF1 expression. Based on these results, we discuss the use of anti-TM4SF4 antibody against TM4SF4-overexpressing and radiation-resistant lung cancer therapy.

## RESULTS

### TM4SF4 is overexpressed in radiation-resistant lung adenocarcinoma A549 cells

**Figure 1:**
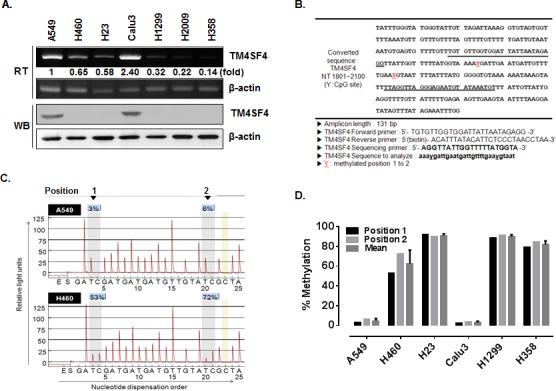
TM4SF4 expression in lung cancer cell lines is regulated by methylation (A) RT-PCR and Western blot analysis of TM4SF4 expression in the indicated lung cancer cell types. Band intensities were measured using Image J software (National Institute of Health, Bethesda, MD, USA), normalized to β-actin and fold increase were indicated. (B) Bisulfite-converted sequence of TM4SF4. Each Y of underlined sequences indicates a methylated position. (C) Pyrosequencing diagram of A549 and H460 cells and comparison of methylation percentages at each CpG position. Each gray box in the diagram indicates the position of the two Y’s shown in panel B. (D) Methylation percentage of *TM4SF4* gene in indicated lung cancer cells.

A549 NSCLC adenocarcinoma cancer cells are more invasive and resistant to radiation than the H460 NSCLC cell line [23, 24]. To identify novel genes involved in radiation resistance of NSCLC cells, expression levels of 30,000 human genes in A549 and H460 cells were compared using DNA microarray analysis. Among hundreds of differentially regulated genes, a dramatic difference in the expression level of TM4SF4 was observed between these cells; A549 cells expressed TM4SF4 at a level approximately 30-fold greater than that observed in H460 cells (data not shown). Based on these results, expression of TM4SF4 in various NSCLC cells, including A549, H460, H23, Calu-3, H1299, H2009 and H358 cells, were analyzed by RT-PCR and Western blotting (Figure 1A). Most of lung cancer cells examined expressed low levels of TM4SF4; however, A549 and Calu-3 cells showed exceptionally high levels of TM4SF4 expression.

A significant difference in gene expression is usually regulated by DNA methylation, a common epigenetic signaling tool that cells use to repress transcription. To examine the regulation of TM4SF4 expression by methylation in the NSCLC cells tested above, puta­tive CpG islands within the promoter and 5′-untranslated region (5′-UTR) of the *TM4SF4* gene were analyzed using the Methprimer program (http://www.urogene.org//methprimer) [25], and two CpG islands were suggested as methylation sites (Figure 1B). In A549 cells, the two positions were methylated less than 10%. In contrast, the *TM4SF4* gene in H460 cells showed a level of methylation greater than 50% (Figure 1C). The methylation percentage of the *TM4SF4* gene was also analyzed in other lung cancer cells. As shown in Figure 1D, lung cancer cells including H23, H1299 and H358, showed high methylation levels, of greater than 80%. However, Calu-3 cells as well as A549 cells showed very low levels, of less than 10% DNA methylation.

In normal lung cells, TM4SF4 is expressed at a very low level [17]. Also, previous studies of TM4SF4 were focused on its functions in intestinal epithelium and liver and showed that TM4SF4 is a negative regulator of cell proliferation [16, 18]. Therefore, TM4SF4 functions in cancer, especially in lung cancer, have not been studied, although other members of the tetraspanin L6 family, such as TM4SF1 and TM4SF5, have been investigated as inducers of tumorigenesis. Based on our findings about the expression and methylation patterns of TM4SF4 in NSCLC cells, we hypothesized that overexpression of TM4SF4 in some aggressive NSCLC adenocarcinoma cells is critical for tumorigenesis and radiation resistance, and we examined this hypothesis using TM4SF4-overexpressing or knockdown A549 NSCLC cells.

### Overexpression of TM4SF4 in A549 cells enhanced cell growth, migration, and invasion

**Figure 2:**
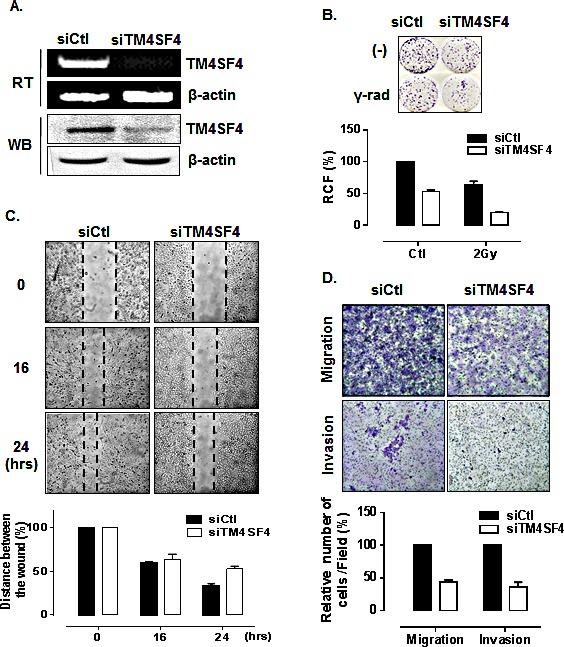
Suppression of TM4SF4 expression in A549 cells retarded cell growth, migration, and invasiveness (A) RT-PCR and Western blot analysis of TM4SF4 expression in A549 cells transfected with siRNA targeting TM4SF4 (siTM4SF4): RNA and proteins were extracted from cells 72 h post-transfection. (B) Colony-forming assay of siTM4SF4-treated A549 cells: 24 h after siRNA transfection, cells were irradiated with a single dose of 2 Gy and, 24 h later, plated for colony-forming assay. Cells were incubated for 10 days and colonies stained with crystal violet were counted, and relative colony forming percentage (RCF) was plotted. (C) Wound-healing assay of siTM4SF4-transfected A549 cells: A549 cells transfected with siTM4SF4 or control siRNA (siCtl) 72 h after transfection were used in assay. At each period of assay, cells were photographed, wound size was determined, and size relative to initial size was represented as plotted. (D) Assay of migration (upper panel) and invasion (lower panel) of siTM4SF4-transfected A549 cells: A549 cells transfected with siTM4SF4 or siCtl were used for assay 48 h after transfection. Twenty-four hours later, cells were stained, photographed under a phase contrast microscope. Number of stained cells were counted and relative number of cells per field was plotted.

First, we analyzed the effect of downregulation of TM4SF4 in A549 cells to examine whether the suppression of TM4SF4 inhibit the tumorigenicity of A549 cells. Short interfering RNA (siRNA)-mediated suppression of TM4SF4 in A549 cells (Figure 2A) retarded cell growth, which was reduced to 50% of control value (Figure 2B). Resistance to gamma irradiation was also decreased severely. Gamma irradiation reduced relative colony formation by A549 cells to 62%, and that of TM4SF4-knockdown cells to 38%, of the level seen in control cells (Figure 2B). To monitor cell migration, a wound healing assay was performed. As shown in Figure 2C, the rate of wound closure was slower in TM4SF4-knockdown cells. Results of transwell migration assay showed the same tendencies (Figure 2D, upper panel). It was also confirmed that TM4SF4 is involved in invasiveness of tumor cells. With suppression of TM4SF4, invasion of A549 cells through Matrigel was significantly decreased (Figure 2D, lower panel).

**Figure 3:**
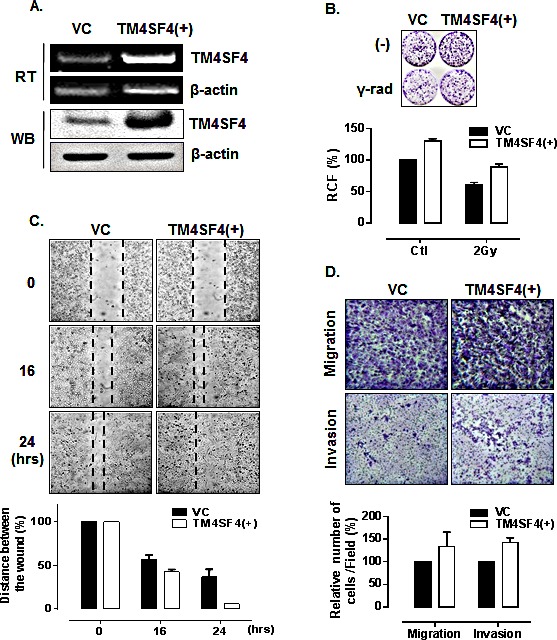
Overexpression of TM4SF4 in A549 cells enhanced cell growth, migration, and invasiveness (A) RT-PCR and Western blot analysis of TM4SF4 expression in pcDNA-TM4SF4-transfected A549 cells: RNA and proteins were extracted from cells 72 h post-transfection. (B) Colony-forming assay of TM4SF4-overexpressing A549 cells: 24 h after transfection, cells were irradiated with a single dose of 2 Gy and, 24 h later, plated for colony-forming assay. (C) Wound-healing assay of TM4SF4-overexpressing A549 cells: A549 cells transfected with pcDNA-TM4SF4 or control vector were used for assay 72 h after transfection. At each period of assay, cells were photographed and wound size was determined. (D) Assay of migration (upper panel) and invasion (lower panel) of TM4SF4-overexpressing A549 cells: A549 cells transfected with pcDNA-TM4SF4 or control vector were used for assay 48 h after transfection.

In contrast, A549 cells transfected with TM4SF4-overexpressing vector, which express TM4SF4 at a higher level than untransfected A549 cells (Figure 3A) showed enhanced cell growth and radiation resistance when compared by colony-forming assay (Figure 3B). The rates of wound closure (Figure 3C) and transwell migration (Figure 3D, upper panel) were also increased by overexpression of TM4SF4. Finally, Matrigel assay with TM4SF4-overexpressing cells showed again that TM4SF4 expression is closely involved in cell invasive ability (Figure 3D, lower panel).

### TM4SF4 is involved in activation of PI3K, AKT, NF-kappaB, and IGF1R

Overexpression of TM4SF4 in the invasive A549 NSCLC adenocarcinoma cell line, and its effects on the cellular characteristics of lung carcinoma cells, revealed that TM4SF4 is critical for tumorigenesis and radiation resistance in A549 NSCLC cells. Calu-3, which is another NSCLC cell possessing the hypomethylated CpG islands of TM4SF4 gene (Figure 1D), also overexpressed TM4SF4 (Figure 1A) and showed similar patterns of cell growth and radiation resistance as A549 cells when expression of TM4SF4 was suppressed or overexpressed (Supplementary Figure 1A).

**Figure 4:**
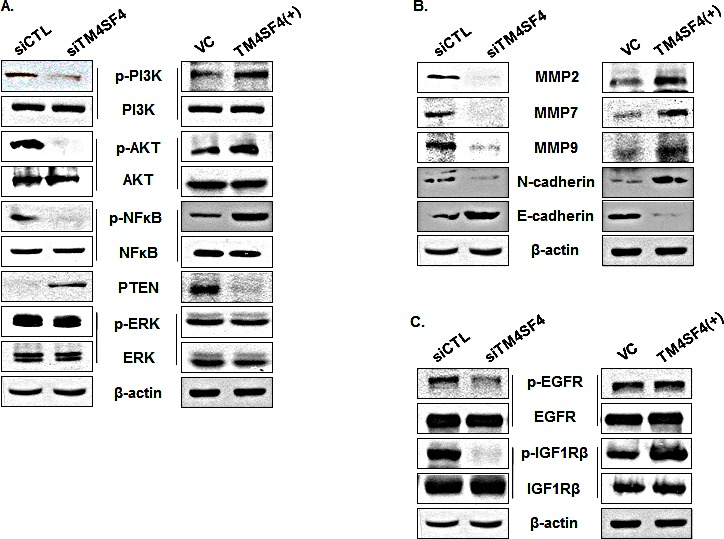
PI3K, AKT, NF-kappaB, PTEN, and MMPs were regulated by TM4SF4 expression in A549 cells (A) Western blot analysis of levels of phosphorylated or total PI3K, AKT, NF-kappaB, and ERK in siTM4SF4- or pcDNA-TM4SF4–transfected A549 cells. PTEN expression was also analyzed. Beta-actin served as internal control. (B) Western blot analysis of metalloproteinases 2, 7, and 9, N-cadherin, and C-cadherin in siTM4SF4- or pcDNA-TM4SF4–transfected A549 cells. (C) Phosphorylation of EGFR and IGF1R in siTM4SF4- and pcDNA-TM4SF4–transfected A549 cells.

To investigate the mechanism of TM4SF4-mediated cell signaling related to tumorigenicity of A549 cells, activations of the PI3K/AKT, NF-kappaB, and ERK signaling pathways, which are involved in cell growth, radiation resistance, migration, and invasion were analyzed. As shown in Figure 4A, suppression of TM4SF4 inhibited activity of the PI3K/AKT pathway. Phosphorylation of AKT and PI3K was severely inhibited, although total levels of AKT and PI3K were not affected. In these cells, PTEN, a phosphatase involved in inhibition of AKT signaling [26], was elevated in correlation with TM4SF4 expression level. Phosphorylation of NF-kappaB was also decreased without an increase in its expression level. However, ERK phosphorylation was not influenced by TM4SF4 expression level. In contrast, overexpression of TM4SF4 elevated phosphorylation of AKT, PI3K, and NF-kappaB. PTEN expression was also completely suppressed by TM4SF4 overexpression. Matrix metalloproteases (MMPs), which are involved in cell migration and invasion, were also analyzed (Figure 4B). MMP-2, 7, and 9 were reduced in TM4SF4-knockdown cells and increased in TM4SF4-overexpressing cells, which is consistent with the effect of TM4SF4 on cellular migration and invasion.

There are several upstream factors which control activation of the PI3K/AKT pathway. In lung cancer cells, IGF1R and EGFR signaling play key roles and have been suggested as major factors upstream of the PI3K/AKT pathway. Phosphorylation of EGFR in A549 cells was inhibited by TM4SF4 knockdown and slightly increased by TM4SF4 overexpression; however, TM4SF4 knockdown severely inhibited phosphorylation of IGF1R (Figure 4C). Overexpression of TM4SF4 also significantly increased the level of phosphorylated IGF1R. These results suggest that TM4SF4 reinforces tumorigenicity of lung cancer cells primarily through the IGF1R signaling pathway.

### TM4SF4 is involved in the activation of IGF1R by up-regulation of IGF1 via NF-κB activation

Most members of TM4SF family mediate signal transduction events related to the regulation of cell development, activation, growth, and motility and these effects have been investigated primarily in the context of their cooperation with integrin signaling within the plasma membrane [27]. TM4SF family proteins also mediate signal transduction events by regulating the expression of growth factors and their receptors [28]. TM4SF4 expression is also related to the expression of several growth factors and receptors, such as TNF-alpha, TNFR1, and c-Met in liver cells [18]. Activation of IGF1R which is essential for the initiation and progression of many cancers [29, 30] starts by ligand-initiated kinase activation with IGF1 or IGF2. Both types of IGF are synthesized in many fetal and adult tissues and secreted IGFs act in autocrine or paracrine manner. However, most of extracellular IGFs are complexed with IGFBP-3, the most prevalent extracellular IGF binding protein, which indicate that free IGFs in serum is important for the activation of IGF1R [31-36].

**Figure 5:**
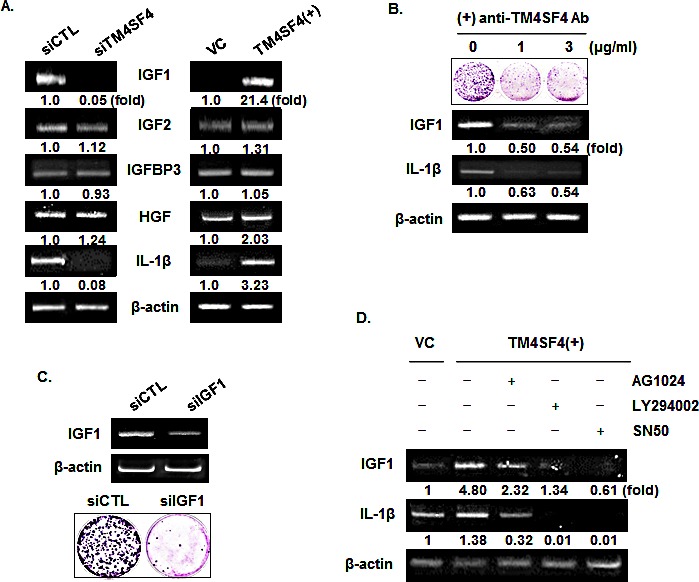
IGF1R activation of TM4SF4-overexpressing A549 cells is induced by enhanced expression of IGF1 via NF-kappaB activation (A) RT-PCR analysis of components of IGF1R signaling pathway and IL1 beta in TM4SF4- knockdown or overexpressing A549 cells. HGF served as negative control and beta-actin as internal control. Band intensities were measured using Image J software, normalized to β-actin and fold increase were indicated. (B) Colony formation assay and RT-PCR analysis of effect of blocking of TM4SF4 by anti-TM4SF4 antibody in TM4SF4-overexpressing A549 cells. (C) Colony formation assay of IGF1-knockdown A549 cells. (D) Effects of IGF1R, PI3K, or NF-kappaB inhibition on the expression of IGF1 and IL1 beta. TM4SF4-overexpressing A549 cells were treated with AG1024 (10 μM for 24 h), LY294002 (50 μM for 48 h) and SN50 (20 μM for 24 h) and analyzed by RT-PCR.

To examine the mechanism of IGF1R activation in TM4SF4-overexpressing A549 cells, we first analyzed the expression level of each component in the IGF1R pathway. As shown in Figure 4C, levels of IGF1R and EGFR were not influenced by knockdown or overexpression of TM4SF4. The transcripts of their specific ligands (IGF1 and IGF2) and their regulatory component (IGFBP-3) were also analyzed by RT-PCR. As shown in Figure 5A, expression of IGF2 and IGFBP3 was not altered by knockdown or overexpression of TM4SF4. The expression of hepatocyte growth factor (HGF), which was analyzed as a growth factor unrelated to the IGF1R signaling pathway, was also unaltered. In contrast, IGF1 expression was decreased or increased about 20-fold, depending on the expression of TM4SF4. These results suggest that IGF1R activation mediated by TM4SF4 overexpression in A549 cells may be the result of regulation of IGF1 expression, specifically, the concentration of free extracellular IGF1. Interaction between TM4SF4 and IGF1R on the plasma membrane was also investigated by co-immunoprecipitation using anti-IGF1R antibody, as in our previous study of IGF1R activation by transgelin [37]; however, direct association of TM4SF4 and IGF1R was not observed (data not shown). In addition, IL1beta expression, which is known to facilitate metastasis of lung cancer [38], was also altered depending on the level of TM4SF4.

TM4SF4 is a membrane protein which can be easily targeted by specific antibody. We examined next whether antibody-mediated inactivation of TM4SF4 suppresses tumor cell growth or the expression of growth factors. Cells were treated with anti-TM4SF4 antibody (1~3μg/mL) and colony-forming units were assessed. As shown in Figure 5B, antibody treatment greatly suppressed colony formation by A549 cells. In these cells, levels of IGF1 and IL1beta transcripts were also decreased, which might cause the decrease in cell growth. Correlation between the decrease of IGF1 and cell growth was also confirmed by colony formation assay of IGF1-knockdown A549 cells (Figure 5C). When TM4SF4-overexpressing Calu-3 cells were treated with anti-TM4SF4 antibody or anti-IGF1 antibody colony formation was reduced also (Supplementary Data 1B).

As shown in Figure 4, overexpression of TM4SF4 activated PI3K, AKT, NF-kappaB, and IGF1R in A549 cells. To confirm signaling molecules related to TM4SF4-mediated induction of IGF1 and IL1beta, TM4SF4-overexpressing A549 cells were treated with the specific inhibitors AG1024 (inhibits IGF1R), LY294002 (inhibits PI3K), SN50 (inhibits NF-kappaB nuclear translocation), and the expression patterns of IGF1 and IL1beta were examined by RT-PCR. As shown in Figure 5D, AG1024 inhibited the expression of IGF1 and IL1beta. Inhibition of PI3K or NF-kappaB also almost completely suppressed the expression of IGF1 and IL1beta, which indicates that TM4SF4-mediated induction of IGF1 requires IGF1R mediated PI3K activation and nuclear translocation of NF-kappaB.

### Antibody-mediated inactivation of TM4SF4 inhibited the growth of xenograft tumors

**Figure 6:**
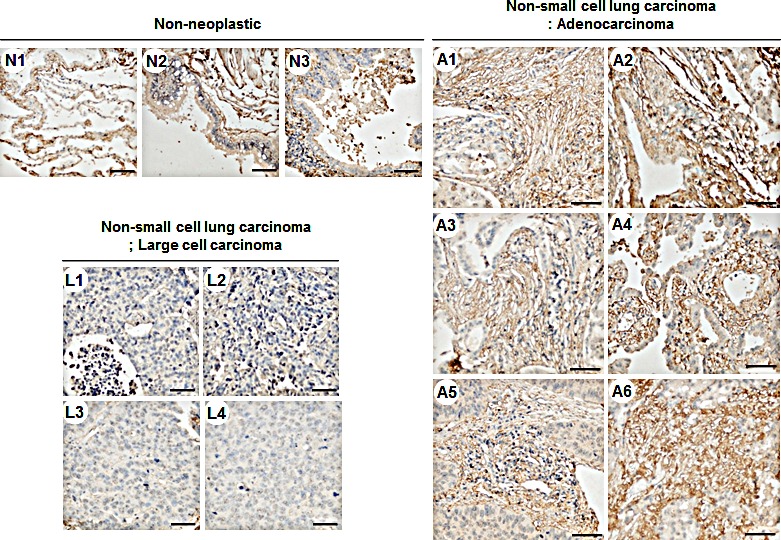
TM4SF4 expression in lung adenocarcinoma but not in large cell carcinoma Non-neoplastic tissue (n=3, N1~N3), large cell carcinoma (n=4, L1~L3), and lung adenocarcinoma (n=6, A1~A6) tissue microarrays were stained with anti-TM4SF4 antibody and secondary reagent (brown). Nuclei were counterstained with hematoxylin (blue). Scale bar 50μm.

We analyzed TM4SF4 levels in A549 NSCLC cells and identified TM4SF4 as an inducer of lung cancer cell tumorigenicity. Moreover, treatment with anti-TM4SF4 antibody was an effective method for suppressing the growth of TM4SF4-overexpressing A549 cells. To evaluate the importance of TM4SF4 in human lung cancer and its potential as a therapeutic target, TM4SF4 expression in lung cancer tissue was confirmed by immunohistochemical staining. As shown in Figure 6, lung adenocarcinoma tissues (A1~A6) were stained dominantly compared to non-neoplastic tissues (N1~N3); however, large cell carcinoma tissues (L1~L4) were not stained. These results suggest that TM4SF4 expression is related to lung adenocarcinoma and it can be used as a biomarker as well as a therapeutic target of lung adenocarcinoma.

**Figure 7:**
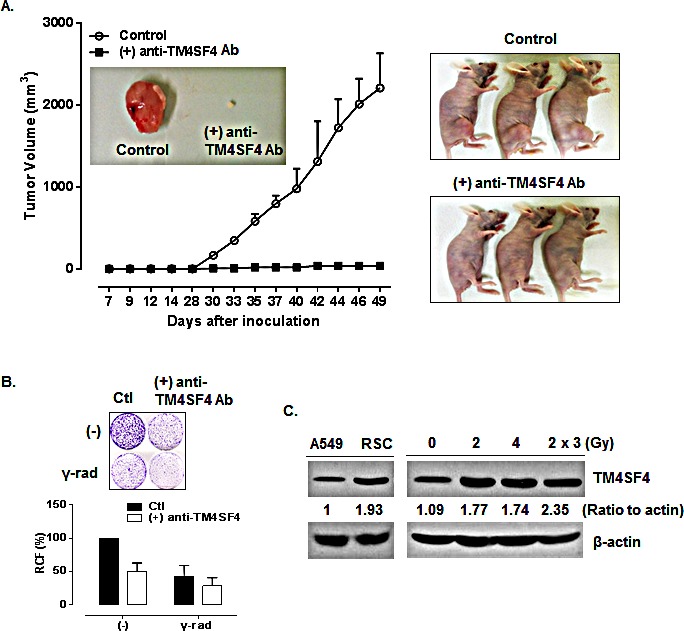
Anti-TM4SF4 antibody treatment inhibited the growth of xenograft tumor established by injection of TM4SF4-overexpressing A549 cells (A) Anti-TM4SF4 antibody was injected into the implantation site of TM4SF4-overexpressing A549 cells at six time points (19, 21, 23, 26 and 28 d) after tumor cell implantation in mice and tumor volume was measured. (B) Colony-forming assay of TM4SF4-overexpressing A549 cells treated with anti-TM4SF4 antibody. Gamma-irradiated cells were also treated with anti-TM4SF4 antibody. Ten days after plating, colonies were counted and relative cell viability was plotted. (C) TM4SF4 expression in gamma-irradiated A549 cells. A549 cells gamma irradiated with a single dose of 2 or 4Gy, or three dose of 2Gy weekly and radiation-selected cells (RSC) after three dose of irradiation were analyzed by Western blot analysis.

The therapeutic efficacy of anti-TM4SF4 antibody was evaluated in mouse xenograft models of TM4SF4-overexpressing human lung adenocarcinomas. When athymic BALB/c nude mice were subcutaneously inoculated with TM4SF4-overexpressing A549 human lung cancer cells, visible tumors were usually detectable 18 days later (Figure 7A). Inoculated mice were divided into two groups, one group in which anti-TM4SF4 antibody was injected peritumorally at six time points after tumor injection, and the control group, in which vehicle (phosphate-buffered saline [PBS]) was injected. As shown in Figure 7A, treatment with anti-TM4SF4 antibody almost completely eliminated the growth of the xenograft tumors, compared with the control group. Without antibody treatment, tumor burdens increased up to 2,500 mm^3^ 49 days after injection of tumor cells; however tumor volume in the anti-TM4SF4 antibody-treated group was less than 100 mm^3^. In addition, anti-TM4SF4 antibody treatment enhanced radiation sensitivity of lung cancer cells. As shown in Figure 7B, gamma irradiation suppressed survival of A549 cells to about 50% that of untreated cells, and treatment with anti-TM4SF4 antibody also effectively suppressed the survival of A549 cells. However, treatment of gamma-irradiated cells with anti-TM4SF4 antibody more severely decreased the tumor cell survival. More importantly, we confirmed that repetitive radiation on A549 lung cancer cells increased the expression of TM4SF4 (Figure 7C). Overall, these results suggest that TM4SF4 is a key protein conferring radiation resistance in lung adenocarcinoma and a combination therapy employing radiation and targeting TM4SF4 might be effective therapy against radiation-resistant lung adenocarcinoma.

## DISCUSSION

In this study, we demonstrated that TM4SF4 is overexpressed in lung adenocarcinoma cells as a result of hypomethylation in the promoter region of the *TM4SF4* gene, and it enhances activation of the IGF1R pathway and, consequently, enhances tumorigenicity. Immunohistochemical staining of human lung cancer tissue also confirmed that TM4SF4 is overexpressed in lung adenocarcinoma and that it can be used as an indicator of lung adenocarcinoma. Overexpression of TM4SF4 in lung cancer cells increased phosphorylation of IGF1R and activated signaling components of the PI3K pathway, the most important signaling pathway in lung cancer cells, including PI3K, AKT, and NK-kappaB. In these lung cancer cells, TM4SF4 overexpression increased the expression of IGF1, a ligand that activates IGF1R, which appears to be a key event in activation of the IGF1R pathway. Suppression of PTEN, an inhibitory phosphatase of the AKT signaling pathway, also enhances activation of the PI3K/AKT signaling pathway in TM4SF4-overexpressing lung cells.

However, at present it is unclear how overexpression of TM4SF4 initiates these events. Previous studies on other tetraspan family proteins have suggested that tetraspanins form complexes with integrins and growth factor receptors to create massive tetraspanin-enriched domains that consequently function in cell adhesion, proliferation, and motility [39, 40]. Further studies on the function of TM4SF4 in lung cancer cells can be started based on the interaction of TM4SF4 with other molecules, such as integrins, growth factor receptors, and cytoplasmic components, which might reveal the mechanism of IGF1 induction. IL1beta expression, which is known to facilitate metastasis of lung cancer [38], is also altered depending on the expression of TM4SF4. Although we did not investigated further about the induction mechanism or action of IL1beta related to tumorigenicity of TM4SF4-overexpressed lung adenocarcinoma cells in this study, NF-kappaB and PI3K signaling, key components regulating the expression of IGF1 in A549 cells, also regulate the expression of IL1beta as shown in results and investigation about its action in lung cancer cells might show another way of cancer treatment.

Tumorigenic activity of TM4SF4 in lung adenocarcinoma cells was dramatically shown by xenograft assay; moreover, the growth of xenograft tumors was effectively suppressed by treatment with anti-TM4SF4 antibody, which suggests that anti-TM4SF4 antibody can be used for anti-cancer therapy of TM4SF4-overexpressing lung adenocarcinoma. In addition, combination treatment of anti-TM4SF4 antibody and radiation was shown to be more effective in tumor removal.

Approximately 50% of all cancer patients receive some type of radiation therapy during the course of their treatment. Patients with lung cancer are also frequently treated with radiation therapy, and more than half of those diagnosed with NSCLC receive radiation therapy. Radiation is used to destroy cancer cells by damaging DNA; however, the DNA can often be repaired, resulting in only temporary responses to treatment. What is worse, in some cases, is that radiation actually increases the expression of cancer-promoting genes, such as *EGFR* [41], resulting in radiation resistance. Therefore, to overcome these situations, many efforts have been made to increase sensitivity or efficiency of radiation therapy. Recently, studies have shown that adding molecularly targeted agents to radiation therapy can prevent repair of radiation-induced damage and thereby improve the treatment response of patients. Drugs that inhibit proteins central to cancer growth or DNA repair, such as the EGFR inhibitor cetuximab, can impede DNA repair and make cancer more susceptible to radiation [41]. In this study, we showed that TM4SF4 can be a therapeutic target in lung cancer. In addition, we demonstrated the potential of combination therapy of targeting TM4SF4 and radiation against lung cancer, although at present this has been confirmed in *in vitro* cell-based assay. Further studies on this combinational therapy *in vivo* and development of novel targeted cancer therapy based on these results may be valuable for treatment of patients with radiation-resistant lung cancer.

## METHODS

### Cell culture

The human lung cancer cell lines A549, H460, H23, Calu-3, H129, H2009, and H358 were obtained from the Korea Cell Line Bank (Seoul, Korea) and grown in RPMI 1640 medium supplemented with 10% (v/v) fetal bovine serum (FBS; Invitrogen, Carlsbad, CA, USA) and 1% penicillin/streptomycin. Cells were incubated at 37°C in a humidified atmosphere of 5% CO_2_.

### DNA methylation analysis

Genomic DNA was extracted from target cells using the DNeasy® Blood & Tissue Kit (Qiagen, Valencia, CA, USA) according to the manufacturer’s protocol and modified by sodium bisulfite using the EZ DNA Methylation-Gold Kit (Zymo Research, Orange, CA, USA) according to the manufacturer’s instructions, as previously described [23, 42].

Pyrosequencing of bisulfite-modified DNA was performed to validate methylation status of candidate loci. Primers for amplification and sequencing were 5′- TGT GTT GGT GGA TTATTAATAGAG G-3′ (forward), 5′-biotin- ACA TTT ATA CAT TCT CCC TAA CCT AA-3′ (reverse) and 5′- AGG TTA TTG GTT TTT ATG GTA-3′ (sequencing). Primers were designed using the Pyrosequencing (PSQ) Assay Design Program (Biotage AB, Uppsala, Sweden) and PCR was carried out as described previously [23]. All primers were obtained from Bioneer (Daejeon, Korea). Pyrosequencing of PCR products was performed using the PyroGold Reagent Kit (Biotage AB) according to the manufacturer’s instruction. Two CpG sites were analyzed and the expected sequence was AAA YGA TTG AAT GAT TGT TTT GAA YGT AAT (Y = T or C, i.e., methylation positions). The methylation percentage at individual CpG sites was then analyzed using the Pyro Q-CpG software (Biotage AB) and the methylation percentage of TM4SF4 was calculated by averaging the methylation percentages at two CpG sites.

### siRNA transfection

A549 cells (1 × 10^5^) were transfected with 50 nM Stealth RNAi^TM^ targeting TM4SF4 (Invitrogen) or Stealth RNAi^TM^ Negative Control Medium GC (Invitrogen) using Lipofectamine^®^ RNAiMAX reagent (Invitrogen). Cells were incubated for 72 h after transfection and then harvested for RT-PCR or western blot analyses. The sequences of Stealth RNAi^TM^ for targeting the *TM4SF4* gene were as follows: sense, 5′-gcc ucu caa ugu ggu ucc cug gaa u-3′ and antisense, 5′-auu cca ggg aac cac auu gag agg c-3′. The sequences of Stealth RNAi^TM^ for targeting the *IGF1* gene were as follows: sense, 5′-CUG UUC ACC AAA UUG UGA A-3′ and antisense, 5′-UUC ACA AUU UGG UGA ACA G-3′.

### Construction and transfection of TM4SF4 overexpression vector

Poly(A) mRNA was isolated from A549 cells, and a 626-bp insert of human *TM4SF4* mRNA was amplified by reverse transcription (RT)-PCR using the following primers: *Eco*RI/forward, 5′- CCA CGA ATT CAT GTG CAC TGG GGG C -3′ and *Xho*I/reverse, 5′- TCC TCG AGT TAA ACG GGT CCA TCT CCC -3′. TM4SF4 cDNA inserts were cloned into the mammalian expression vector pcDNA3.1 (Invitrogen), and the resultant expression vector (pcTM4SF4) was transfected into A549 cells using Lipofectamine^®^ 2000 (Invitrogen).

### Colony-forming assay

Cells were plated in 35-mm culture dishes at a density of 2 × 10^3^ cells per plate, incubated for 10 days, and stained with 0.5% crystal violet. Colonies, defined as groups of ≥50 cells, were then counted and relative colony-forming percentage was plotted. For gamma-irradiation, cells were cultured in T25 flasks at a density of 1 × 10^6^ cells/flask and, after 24 h, irradiated by exposure to a single dose of 20 Gy (^60^Co gamma ray source; dose rate, 2 Gy/min). Then the cells were plated for colony-forming assay. To inactivate TM4SF4 on the surface of A549 cells, anti-TM4SF4 antibody was added to medium used for the colony-forming assay at concentration of 1 or 3 μg/ml.

### Wound-healing assay

A549 cells were transfected with TM4SF4-specific siRNA or TM4SF4 overexpression vector. After 72 h, cells were re-plated in 35-mm culture dishes and cultured until the monolayer was 100% confluent. Cells were then serum-starved overnight by incubation in RPMI 1640 medium containing 0.5% FBS, followed by introduction of a scratch wound with a plastic pipette tip. Medium was replaced with fresh growth medium, and images were taken at regular intervals over the course of 12–36 h. Quantification of cell migration was carried out by measuring the distances between 10 randomly selected points within the wound edge and the mean values with standard deviation were plotted.

### Migration assay

Untransfected control A549 cells or A549 cells 48 h after transfection with pcTM4SF4 or TM4SF4-specific siRNA were analyzed in a transwell migration assay. In brief, the lower culture chamber of a 24-transwell plate (Cell Biolabs, San Diego, CA, USA) was filled with 500 μL migration medium, consisting of RPMI 1640 and 10% FBS. Cells were seeded in the upper chamber, at a density of 2 × 10^5^ cells in 300 μl serum-free medium/well, and incubated for 24 h at 37°C in a humidified atmosphere of 5% CO_2_. Non-migratory cells in the upper chamber were removed by wiping with a cotton swab. Migratory cells on the bottom of chambers were stained with crystal violet, and cells were counted under a light microscope.

### Invasion assay

Cell invasion was determined using Matrigel-coated invasion chambers (8-μm pores; BD Biosciences, Bedford, MA, USA) according to the manufacturer′s instructions. Untransfected control A549 cells or A549 cells 48 h after transfection with pcTM4SF4 or TM4SF4-specific siRNA were incubated for 24 h in serum-free RPMI 1640 and then detached from the cell culture plates using a non-enzymatic cell dissociation solution (Sigma-Aldrich, St Louis, MO, USA). Cells were resuspended in serum-free RPMI 1640, placed in the upper invasion chamber at a density of 5 × 10^4^ cells/well, and RPMI 1640 containing 10% (v/v) FBS was added to the lower chamber. The plates were incubated in a at 37°C in a humidified atmosphere of 5% CO_2_ for 24 h, and noninvasive cells in the upper chamber were removed by wiping with a cotton swab. The invasive cells in the underside of inserts were fixed with 4% (w/v) formaldehyde in phosphate-buffered saline and stained with 2% (w/v) crystal violet in 2% (v/v) ethanol. The stained cells that had penetrated the Matrigel were counted under a light microscope.

### cDNA synthesis and PCR amplification

Total RNA was isolated from cells with TRIzol reagent (Invitrogen) following the manufacturer’s instructions. First-strand cDNA was generated from 1 μg of total RNA using oligo dT primers and a cDNA synthesis kit (Intron Biotechnology, Gyungki-do, Korea). Resultant cDNA served as templates for PCR amplification with the following primers: TM4SF4 forward, 5′-CCA CGA ATT CAT GTG CAC TGG GGG C-3′ and reverse, 5′-TCC TCG AGT TAA ACG GGT CCA TCT CCC-3′; beta-actin forward, 5′-CAT CCT CAC CCT GAA GTA CCC-3′ and reverse, 5′-AGC CTG GAT AGC AAC GTA CAT G-3′. PCR was carried out under conditions of initial denaturation at 94°C for 5 min; followed by 30 cycles of 94°C for 1 min, 59°C for 30 sec, and 72°C for 1 min; and a final extension at 72°C for 5 min. The amplified PCR products were separated on 1% agarose gels (Intron Biotechnology) and stained with ethidium bromide.

### Western blot analysis

Anti-TM4SF4 antibody for western blot analysis was purchased from Sigma-Aldrich. Antibodies against phospho-Ser473 AKT, AKT, phospho-ERK, ERK, phospho-NF-kappaB (p65), NF-kappaB, phospho-PI3K, PI3K, PTEN, phospho-Tyr1068 EGFR, EGFR, phospho-IGF1R beta (Tyr1131)/insulin receptor beta (Tyr1146), IGF-1R beta (111A9), and anti-beta-actin antibodies were purchased from Cell Signaling Technology (Danvers, MA, USA). Anti-MMP-2, anti-MMP-7, and anti-MMP-9 antibodies were purchased from Santa Cruz Biotechnology (Santa Cruz, CA, USA). Western blot analysis was performed as described previously [37]. Antibodies against N-cadherin and E-cadherin were purchased from BD Biosciences.

**Table 1:** RT-PCR primer sequences

Target	Primer sequence
TM4SF4	F: 5′-CCA CGA ATT CAT GTG CAC TGG GGG C-3′
	R: 5′-TCC TCG AGT TAA ACG GGT CCA TCT CCC-3′
IGF1	F: 5′-TGC TCA CCT TCA CCA GCT CTG CCA-3′
	R: 5′-GTG TGG CGC TGG GCA GGG ACA GA-3′
IGF2	F: 5′-GGT ACC ATC GAA TGG CGG GGT-3′
	R: 5′-CTC GAG TTA CTC GGA CTT GGC-3′
IGFBP3	F: 5′-CAG TAC GTC GCC CGC GCT GGG-3′
	R: 5′-CGT CTA CTT GCT CTG CAT GCT-3′
HGF	F: 5′-CCA TGA TAC CAC ACG AAC ACA GC-3′
	R: 5′-GTC AAG AGT ATA GCA CCA TGG CCT-3′
IL1 beta	F: 5′-ATG GCA GAA GTA CCT AAG CTC GC-3′
	R: 5′-TTG ATC GAA GTG GTA CGT TAA ACA CA-3′
Beta-actin	F: 5′-CAT CCT CAC CCT GAA GTA CCC-3′
	R: 5′-AGC CTG GAT AGC AAC GTA CAT G-3′

F, forward; R, reverse

### Xenograft tumor growth assay

Tumorigenicity of TM4SF4-overexpressing cells was estimated by xenograft assay. A549 cells (5 × 10^5^ cells per injection) overexpressing TM4SF4 were resuspended in 100 μL PBS and subcutaneously inoculated into the flanks of 5-week old athymic BALB/c female nude mice (n=5 per group). At 3-day intervals after tumor cell injection, tumor size was measured using calipers (calculated volume=shortest diameter^2^×longest diameter/2). Seven weeks after cell inoculation, the grafts were removed and photographed. To evaluate the antitumor efficacy of anti-TM4SF4 antibody treatment on lung cancer, two groups of xenograft tumor mice inoculated with TM4SF4-overexpressing A549 cells were prepared and anti-TM4SF4 antibody (7 μg/18 g body weight) was injected into the tumor inoculation site at six time points (19, 21, 23, 26, 28, and 30 d after tumor cell injection) after the tumor burdens became measurable. The control group was injected with PBS. Tumor size was measured at 3-day intervals; 6 weeks after inoculation, the grafts were removed and photographed. All animal studies were approved by the institutional review board (KRIBB Institutional Animal Care and Use Committee/KRIBB-IACUC, approval number: KRIBB-AEC-4253), and all procedures were performed in accordance with institutional guidelines for animal care.

### Immunohistochemistry

Lung cancer tissue sections (AccuMax Array; ISU ABXIS Co, Seongnam, Gyeonggi, Korea) were heated at 60°C for 30 min. Sections were deparaffinized and rehydrated by incubation in three changes of xylene (3 min each), three changes of 100% ethanol (2 min each), 95% ethanol for 2 min, 85% ethanol for 2 min, 75 % ethanol for 2 min, and two changes of distilled water. For antigen retrieval, sections were incubated in 0.3 % hydrogen peroxide at room temperature for 30 min to inactivate endogenous peroxidase, followed by three washes in PBS. Tissue sections were incubated with anti-TM4SF4 polyclonal antibody (1:100; Sigma-Aldrich) and incubated for 1 h at room temperature. For a negative control, the primary antibody was replaced with PBS. After three washes with PBS, sections were incubated with secondary reagent (Vectastain ABC Immunohistochemistry Kit for rabbit; Vector Laboratories, Burlingame, CA, USA). Sections were washed with water to stop the reaction. Sections were counterstained with hematoxylin, rehydrated by passage through a graded series of alcohols (100%, 95%, 85%, and 75%), and mounted with xylene.

### Statistical analysis

Each experiment was repeated three times, and a representative result was shown. Statistical analysis was performed using PRISM version 5.0 (GraphPad, San Diego, CA, USA). Data are presented as the mean±standard deviation (SD) and error bar represent standard deviation.

## SUPPLEMENTARY FIGURE


