# Supplementary material for: TM4SF4 overexpression in radiation-resistant lung carcinoma cells activates IGF1R via elevation of IGF1
Source: Oncotarget. 2014 Sep 8;5(20):9823–37. doi: 10.18632/oncotarget.2450 (PMC4259440; doi:10.18632/oncotarget.2450)
Supplement: Supplementary file 1 [file oncotarget-05-9823-s001.pdf]

TM4SF4 overexpression in radiation-resistant lung carcinoma cells activates IGF1R via elevation of IGF1

Supplementary Material

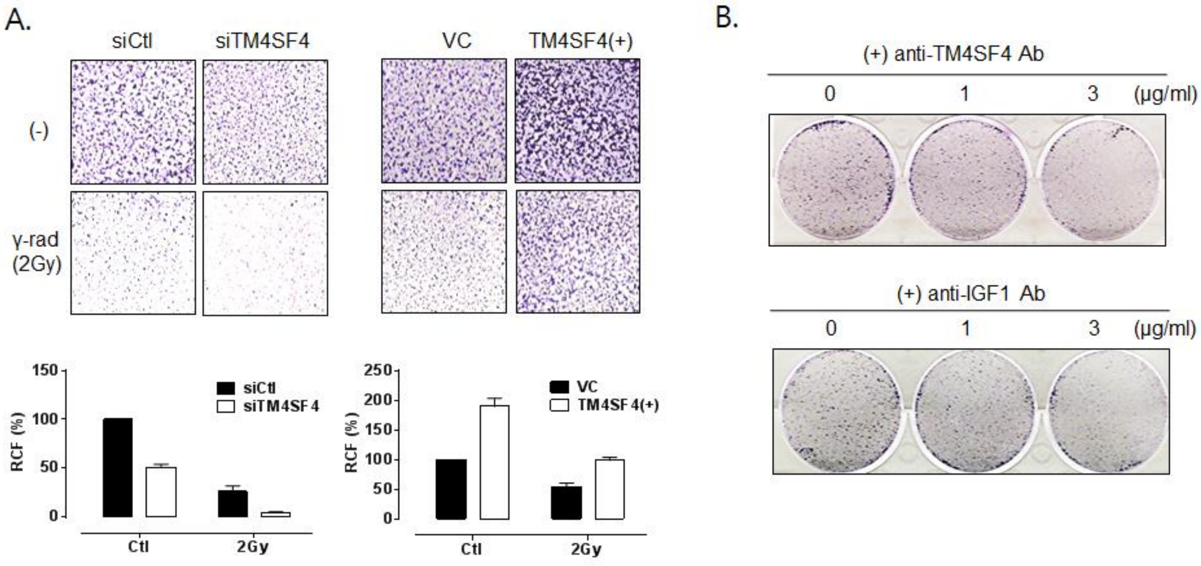

Supplementary Figure 1: **TM4SF4-suppressiong or overexpressing Calu-3 cells.** (A) Colony-forming assay of siTM4SF4- or pcDNA-TM4SF4 transfected Calu-3 cells. 24 h after transfection, cells were irradiated with a single dose of 2 Gy and, 24 h later, plated for colony-forming assay. Cells were incubated for 10 days and colonies stained with crystal violet were counted, and relative colony forming percentage (RCF) was plotted. (B) Colony formation assay of TM4SF4-overexpressing A549 cells treated with anti-TM4SF4 antibody or anti-IGF1 antibody.
